# Supplementary material for: Resolving the full spectrum of human genome variation using Linked-Reads
Source: Genome Res. 2019 Apr;29(4):635–45. doi: 10.1101/gr.234443.118 (PMC6442396; doi:10.1101/gr.234443.118)
Supplement: Supplemental Material [file supp_gr.234443.118_Supplemental_longranger-2.2.zip › longranger-2.2.tar.bz2!/lr2.2-public/lib/stan/cmdstan/stan/doc/api/html/classstan_1_1io_1_1reader.html]

Stan: stan::io::reader< T > Class Template Reference


|  |  |
| --- | --- |
|  | Stan  2.5.0  probability, sampling & optimization |


- Main Page
- Related Pages
- Namespaces
- Classes
- Files

- Class List
- Class Index
- Class Hierarchy
- Class Members

All Classes Namespaces Files Functions Variables Typedefs Enumerations Enumerator Friends Macros Pages

- stan
- io
- reader

Public Types |
Public Member Functions |
List of all members

stan::io::reader< T > Class Template Reference

A stream-based reader for integer, scalar, vector, matrix and array data types, with Jacobian calculations.
More...

`#include <reader.hpp>`

|  |  |
| --- | --- |
| Public Types | |
| typedef Eigen::Matrix< T,   Eigen::Dynamic, Eigen::Dynamic > | matrix\_t |
|  | |
| typedef Eigen::Matrix< T,   Eigen::Dynamic, 1 > | vector\_t |
|  | |
| typedef Eigen::Matrix< T,   1, Eigen::Dynamic > | row\_vector\_t |
|  | |
| typedef Eigen::Map< matrix\_t > | map\_matrix\_t |
|  | |
| typedef Eigen::Map< vector\_t > | map\_vector\_t |
|  | |
| typedef Eigen::Map< row\_vector\_t > | map\_row\_vector\_t |
|  | |

|  |  |
| --- | --- |
| Public Member Functions | |
|  | reader (std::vector< T > &data\_r, std::vector< int > &data\_i) |
|  | Construct a variable reader using the specified vectors as the source of scalar and integer values for data. More... |
|  | |
|  | ~reader () |
|  | Destroy this variable reader. More... |
|  | |
| size\_t | available () |
|  | Return the number of scalars remaining to be read. More... |
|  | |
| size\_t | available\_i () |
|  | Return the number of integers remaining to be read. More... |
|  | |
| int | integer () |
|  | Return the next integer in the integer sequence. More... |
|  | |
| int | integer\_constrain () |
|  | Return the next integer in the integer sequence. More... |
|  | |
| int | integer\_constrain (T &) |
|  | Return the next integer in the integer sequence. More... |
|  | |
| T | scalar () |
|  | Return the next scalar in the sequence. More... |
|  | |
| T | scalar\_constrain () |
|  | Return the next scalar. More... |
|  | |
| T | scalar\_constrain (T &) |
|  | Return the next scalar in the sequence, incrementing the specified reference with the log absolute Jacobian determinant. More... |
|  | |
| std::vector< T > | std\_vector (size\_t m) |
|  | Return a standard library vector of the specified dimensionality made up of the next scalars. More... |
|  | |
| vector\_t | vector (size\_t m) |
|  | Return a column vector of specified dimensionality made up of the next scalars. More... |
|  | |
| vector\_t | vector\_constrain (size\_t m) |
|  | Return a column vector of specified dimensionality made up of the next scalars. More... |
|  | |
| vector\_t | vector\_constrain (size\_t m, T &) |
|  | Return a column vector of specified dimensionality made up of the next scalars. More... |
|  | |
| row\_vector\_t | row\_vector (size\_t m) |
|  | Return a row vector of specified dimensionality made up of the next scalars. More... |
|  | |
| row\_vector\_t | row\_vector\_constrain (size\_t m) |
|  | Return a row vector of specified dimensionality made up of the next scalars. More... |
|  | |
| row\_vector\_t | row\_vector\_constrain (size\_t m, T &) |
|  | Return a row vector of specified dimensionality made up of the next scalars. More... |
|  | |
| matrix\_t | matrix (size\_t m, size\_t n) |
|  | Return a matrix of the specified dimensionality made up of the next scalars arranged in column-major order. More... |
|  | |
| matrix\_t | matrix\_constrain (size\_t m, size\_t n) |
|  | Return a matrix of the specified dimensionality made up of the next scalars arranged in column-major order. More... |
|  | |
| matrix\_t | matrix\_constrain (size\_t m, size\_t n, T &) |
|  | Return a matrix of the specified dimensionality made up of the next scalars arranged in column-major order. More... |
|  | |
| int | integer\_lb (int lb) |
|  | Return the next integer, checking that it is greater than or equal to the specified lower bound. More... |
|  | |
| int | integer\_lb\_constrain (int lb) |
|  | Return the next integer, checking that it is greater than or equal to the specified lower bound. More... |
|  | |
| int | integer\_lb\_constrain (int lb, T &) |
|  | Return the next integer, checking that it is greater than or equal to the specified lower bound. More... |
|  | |
| int | integer\_ub (int ub) |
|  | Return the next integer, checking that it is less than or equal to the specified upper bound. More... |
|  | |
| int | integer\_ub\_constrain (int ub) |
|  | Return the next integer, checking that it is less than or equal to the specified upper bound. More... |
|  | |
| int | integer\_ub\_constrain (int ub, T &) |
|  | Return the next integer, checking that it is less than or equal to the specified upper bound. More... |
|  | |
| int | integer\_lub (int lb, int ub) |
|  | Return the next integer, checking that it is less than or equal to the specified upper bound. More... |
|  | |
| int | integer\_lub\_constrain (int lb, int ub) |
|  | Return the next integer, checking that it is less than or equal to the specified upper bound. More... |
|  | |
| int | integer\_lub\_constrain (int lb, int ub, T &) |
|  | Return the next integer, checking that it is less than or equal to the specified upper bound. More... |
|  | |
| T | scalar\_pos () |
|  | Return the next scalar, checking that it is positive. More... |
|  | |
| T | scalar\_pos\_constrain () |
|  | Return the next scalar, transformed to be positive. More... |
|  | |
| T | scalar\_pos\_constrain (T &lp) |
|  | Return the next scalar transformed to be positive, incrementing the specified reference with the log absolute determinant of the Jacobian. More... |
|  | |
| template<typename TL > | |
| T | scalar\_lb (const TL lb) |
|  | Return the next scalar, checking that it is greater than or equal to the specified lower bound. More... |
|  | |
| template<typename TL > | |
| T | scalar\_lb\_constrain (const TL lb) |
|  | Return the next scalar transformed to have the specified lower bound. More... |
|  | |
| template<typename TL > | |
| T | scalar\_lb\_constrain (const TL lb, T &lp) |
|  | Return the next scalar transformed to have the specified lower bound, incrementing the specified reference with the log of the absolute Jacobian determinant of the transform. More... |
|  | |
| template<typename TU > | |
| T | scalar\_ub (TU ub) |
|  | Return the next scalar, checking that it is less than or equal to the specified upper bound. More... |
|  | |
| template<typename TU > | |
| T | scalar\_ub\_constrain (const TU ub) |
|  | Return the next scalar transformed to have the specified upper bound. More... |
|  | |
| template<typename TU > | |
| T | scalar\_ub\_constrain (const TU ub, T &lp) |
|  | Return the next scalar transformed to have the specified upper bound, incrementing the specified reference with the log of the absolute Jacobian determinant of the transform. More... |
|  | |
| template<typename TL , typename TU > | |
| T | scalar\_lub (const TL lb, const TU ub) |
|  | Return the next scalar, checking that it is between the specified lower and upper bound. More... |
|  | |
| template<typename TL , typename TU > | |
| T | scalar\_lub\_constrain (const TL lb, const TU ub) |
|  | Return the next scalar transformed to be between the specified lower and upper bounds. More... |
|  | |
| template<typename TL , typename TU > | |
| T | scalar\_lub\_constrain (TL lb, TU ub, T &lp) |
|  | Return the next scalar transformed to be between the the specified lower and upper bounds. More... |
|  | |
| T | prob () |
|  | Return the next scalar, checking that it is a valid value for a probability, between 0 (inclusive) and 1 (inclusive). More... |
|  | |
| T | prob\_constrain () |
|  | Return the next scalar transformed to be a probability between 0 and 1. More... |
|  | |
| T | prob\_constrain (T &lp) |
|  | Return the next scalar transformed to be a probability between 0 and 1, incrementing the specified reference with the log of the absolute Jacobian determinant. More... |
|  | |
| T | corr () |
|  | Return the next scalar, checking that it is a valid value for a correlation, between -1 (inclusive) and 1 (inclusive). More... |
|  | |
| T | corr\_constrain () |
|  | Return the next scalar transformed to be a correlation between -1 and 1. More... |
|  | |
| T | corr\_constrain (T &lp) |
|  | Return the next scalar transformed to be a (partial) correlation between -1 and 1, incrementing the specified reference with the log of the absolute Jacobian determinant. More... |
|  | |
| vector\_t | unit\_vector (size\_t k) |
|  | Return a unit\_vector of the specified size made up of the next scalars. More... |
|  | |
| Eigen::Matrix< T,   Eigen::Dynamic, 1 > | unit\_vector\_constrain (size\_t k) |
|  | Return the next unit\_vector transformed vector of the specified length. More... |
|  | |
| vector\_t | unit\_vector\_constrain (size\_t k, T &lp) |
|  | Return the next unit\_vector of the specified size (using one fewer unconstrained scalars), incrementing the specified reference with the log absolute Jacobian determinant. More... |
|  | |
| vector\_t | simplex (size\_t k) |
|  | Return a simplex of the specified size made up of the next scalars. More... |
|  | |
| Eigen::Matrix< T,   Eigen::Dynamic, 1 > | simplex\_constrain (size\_t k) |
|  | Return the next simplex transformed vector of the specified length. More... |
|  | |
| vector\_t | simplex\_constrain (size\_t k, T &lp) |
|  | Return the next simplex of the specified size (using one fewer unconstrained scalars), incrementing the specified reference with the log absolute Jacobian determinant. More... |
|  | |
| vector\_t | ordered (size\_t k) |
|  | Return the next vector of specified size containing values in ascending order. More... |
|  | |
| vector\_t | ordered\_constrain (size\_t k) |
|  | Return the next ordered vector of the specified length. More... |
|  | |
| vector\_t | ordered\_constrain (size\_t k, T &lp) |
|  | Return the next ordered vector of the specified size, incrementing the specified reference with the log absolute Jacobian of the determinant. More... |
|  | |
| vector\_t | positive\_ordered (size\_t k) |
|  | Return the next vector of specified size containing positive values in ascending order. More... |
|  | |
| vector\_t | positive\_ordered\_constrain (size\_t k) |
|  | Return the next positive ordered vector of the specified length. More... |
|  | |
| vector\_t | positive\_ordered\_constrain (size\_t k, T &lp) |
|  | Return the next positive\_ordered vector of the specified size, incrementing the specified reference with the log absolute Jacobian of the determinant. More... |
|  | |
| matrix\_t | cholesky\_factor (size\_t M, size\_t N) |
|  | Return the next Cholesky factor with the specified dimensionality, reading it directly without transforms. More... |
|  | |
| matrix\_t | cholesky\_factor\_constrain (size\_t M, size\_t N) |
|  | Return the next Cholesky factor with the specified dimensionality, reading from an unconstrained vector of the appropriate size. More... |
|  | |
| matrix\_t | cholesky\_factor\_constrain (size\_t M, size\_t N, T &lp) |
|  | Return the next Cholesky factor with the specified dimensionality, reading from an unconstrained vector of the appropriate size, and increment the log probability reference with the log Jacobian adjustment for the transform. More... |
|  | |
| matrix\_t | cholesky\_corr (size\_t K) |
|  | Return the next Cholesky factor for a correlation matrix with the specified dimensionality, reading it directly without transforms. More... |
|  | |
| matrix\_t | cholesky\_corr\_constrain (size\_t K) |
|  | Return the next Cholesky factor for a correlation matrix with the specified dimensionality, reading from an unconstrained vector of the appropriate size. More... |
|  | |
| matrix\_t | cholesky\_corr\_constrain (size\_t K, T &lp) |
|  | Return the next Cholesky factor for a correlation matrix with the specified dimensionality, reading from an unconstrained vector of the appropriate size, and increment the log probability reference with the log Jacobian adjustment for the transform. More... |
|  | |
| matrix\_t | cov\_matrix (size\_t k) |
|  | Return the next covariance matrix with the specified dimensionality. More... |
|  | |
| matrix\_t | cov\_matrix\_constrain (size\_t k) |
|  | Return the next covariance matrix of the specified dimensionality. More... |
|  | |
| matrix\_t | cov\_matrix\_constrain (size\_t k, T &lp) |
|  | Return the next covariance matrix of the specified dimensionality, incrementing the specified reference with the log absolute Jacobian determinant. More... |
|  | |
| matrix\_t | corr\_matrix (size\_t k) |
|  | Returns the next correlation matrix of the specified dimensionality. More... |
|  | |
| matrix\_t | corr\_matrix\_constrain (size\_t k) |
|  | Return the next correlation matrix of the specified dimensionality. More... |
|  | |
| matrix\_t | corr\_matrix\_constrain (size\_t k, T &lp) |
|  | Return the next correlation matrix of the specified dimensionality, incrementing the specified reference with the log absolute Jacobian determinant. More... |
|  | |
| template<typename TL > | |
| vector\_t | vector\_lb (const TL lb, size\_t m) |
|  | |
| template<typename TL > | |
| vector\_t | vector\_lb\_constrain (const TL lb, size\_t m) |
|  | |
| template<typename TL > | |
| vector\_t | vector\_lb\_constrain (const TL lb, size\_t m, T &lp) |
|  | |
| template<typename TL > | |
| row\_vector\_t | row\_vector\_lb (const TL lb, size\_t m) |
|  | |
| template<typename TL > | |
| row\_vector\_t | row\_vector\_lb\_constrain (const TL lb, size\_t m) |
|  | |
| template<typename TL > | |
| row\_vector\_t | row\_vector\_lb\_constrain (const TL lb, size\_t m, T &lp) |
|  | |
| template<typename TL > | |
| matrix\_t | matrix\_lb (const TL lb, size\_t m, size\_t n) |
|  | |
| template<typename TL > | |
| matrix\_t | matrix\_lb\_constrain (const TL lb, size\_t m, size\_t n) |
|  | |
| template<typename TL > | |
| matrix\_t | matrix\_lb\_constrain (const TL lb, size\_t m, size\_t n, T &lp) |
|  | |
| template<typename TU > | |
| vector\_t | vector\_ub (const TU ub, size\_t m) |
|  | |
| template<typename TU > | |
| vector\_t | vector\_ub\_constrain (const TU ub, size\_t m) |
|  | |
| template<typename TU > | |
| vector\_t | vector\_ub\_constrain (const TU ub, size\_t m, T &lp) |
|  | |
| template<typename TU > | |
| row\_vector\_t | row\_vector\_ub (const TU ub, size\_t m) |
|  | |
| template<typename TU > | |
| row\_vector\_t | row\_vector\_ub\_constrain (const TU ub, size\_t m) |
|  | |
| template<typename TU > | |
| row\_vector\_t | row\_vector\_ub\_constrain (const TU ub, size\_t m, T &lp) |
|  | |
| template<typename TU > | |
| matrix\_t | matrix\_ub (const TU ub, size\_t m, size\_t n) |
|  | |
| template<typename TU > | |
| matrix\_t | matrix\_ub\_constrain (const TU ub, size\_t m, size\_t n) |
|  | |
| template<typename TU > | |
| matrix\_t | matrix\_ub\_constrain (const TU ub, size\_t m, size\_t n, T &lp) |
|  | |
| template<typename TL , typename TU > | |
| vector\_t | vector\_lub (const TL lb, const TU ub, size\_t m) |
|  | |
| template<typename TL , typename TU > | |
| vector\_t | vector\_lub\_constrain (const TL lb, const TU ub, size\_t m) |
|  | |
| template<typename TL , typename TU > | |
| vector\_t | vector\_lub\_constrain (const TL lb, const TU ub, size\_t m, T &lp) |
|  | |
| template<typename TL , typename TU > | |
| row\_vector\_t | row\_vector\_lub (const TL lb, const TU ub, size\_t m) |
|  | |
| template<typename TL , typename TU > | |
| row\_vector\_t | row\_vector\_lub\_constrain (const TL lb, const TU ub, size\_t m) |
|  | |
| template<typename TL , typename TU > | |
| row\_vector\_t | row\_vector\_lub\_constrain (const TL lb, const TU ub, size\_t m, T &lp) |
|  | |
| template<typename TL , typename TU > | |
| matrix\_t | matrix\_lub (const TL lb, const TU ub, size\_t m, size\_t n) |
|  | |
| template<typename TL , typename TU > | |
| matrix\_t | matrix\_lub\_constrain (const TL lb, const TU ub, size\_t m, size\_t n) |
|  | |
| template<typename TL , typename TU > | |
| matrix\_t | matrix\_lub\_constrain (const TL lb, const TU ub, size\_t m, size\_t n, T &lp) |
|  | |

## Detailed Description

### template<typename T> class stan::io::reader< T >

A stream-based reader for integer, scalar, vector, matrix and array data types, with Jacobian calculations.

The template parameter `T` represents the type of scalars and the values in vectors and matrices. The only requirement on the template type `T` is that a double can be copied into it, as in

`T t = 0.0;`

This includes `double` itself and the reverse-mode algorithmic differentiation class `stan::agrad::var`.

For transformed values, the scalar type parameter `T` must support the transforming operations, such as `exp(x)` for positive-bounded variables. It must also support equality and inequality tests with `double` values.

Template Parameters
:   |  |  |
    | --- | --- |
    | T | Basic scalar type. |

Definition at line 34 of file reader.hpp.

## Member Typedef Documentation

template<typename T >

typedef Eigen::Map<matrix\_t> stan::io::reader< T >::map\_matrix\_t

Definition at line 67 of file reader.hpp.

template<typename T >

typedef Eigen::Map<row\_vector\_t> stan::io::reader< T >::map\_row\_vector\_t

Definition at line 69 of file reader.hpp.

template<typename T >

typedef Eigen::Map<vector\_t> stan::io::reader< T >::map\_vector\_t

Definition at line 68 of file reader.hpp.

template<typename T >

typedef Eigen::Matrix<T,Eigen::Dynamic,Eigen::Dynamic> stan::io::reader< T >::matrix\_t

Definition at line 63 of file reader.hpp.

template<typename T >

typedef Eigen::Matrix<T,1,Eigen::Dynamic> stan::io::reader< T >::row\_vector\_t

Definition at line 65 of file reader.hpp.

template<typename T >

typedef Eigen::Matrix<T,Eigen::Dynamic,1> stan::io::reader< T >::vector\_t

Definition at line 64 of file reader.hpp.

## Constructor & Destructor Documentation

template<typename T >

|  |  |  |  |
| --- | --- | --- | --- |
| stan::io::reader< T >::reader | ( | std::vector< T > & | *data\_r*, |
|  |  | std::vector< int > & | *data\_i* |
|  | ) |  |  |

inline

Construct a variable reader using the specified vectors as the source of scalar and integer values for data.

This class holds a reference to the specified data vectors.

Attempting to read beyond the end of the data or integer value sequences raises a runtime exception.

Parameters
:   |  |  |
    | --- | --- |
    | data\_r | Sequence of scalar values. |
    | data\_i | Sequence of integer values. |

Definition at line 83 of file reader.hpp.

template<typename T >

|  |  |  |  |  |
| --- | --- | --- | --- | --- |
| stan::io::reader< T >::~reader | ( |  | ) |  |

inline

Destroy this variable reader.

Definition at line 94 of file reader.hpp.

## Member Function Documentation

template<typename T >

|  |  |  |  |  |
| --- | --- | --- | --- | --- |
| size\_t stan::io::reader< T >::available | ( |  | ) |  |

inline

Return the number of scalars remaining to be read.

Returns
:   Number of scalars left to read.

Definition at line 101 of file reader.hpp.

template<typename T >

|  |  |  |  |  |
| --- | --- | --- | --- | --- |
| size\_t stan::io::reader< T >::available\_i | ( |  | ) |  |

inline

Return the number of integers remaining to be read.

Returns
:   Number of integers left to read.

Definition at line 110 of file reader.hpp.

template<typename T >

|  |  |  |  |  |  |
| --- | --- | --- | --- | --- | --- |
| matrix\_t stan::io::reader< T >::cholesky\_corr | ( | size\_t | *K* | ) |  |

inline

Return the next Cholesky factor for a correlation matrix with the specified dimensionality, reading it directly without transforms.

Parameters
:   |  |  |
    | --- | --- |
    | K | Rows and columns of Cholesky factor |

Returns
:   Next Cholesky factor for a correlation matrix.

Exceptions
:   |  |  |
    | --- | --- |
    | std::domain\_error | if the matrix is not a valid Cholesky factor for a correlation matrix. |

Definition at line 1010 of file reader.hpp.

template<typename T >

|  |  |  |  |  |  |
| --- | --- | --- | --- | --- | --- |
| matrix\_t stan::io::reader< T >::cholesky\_corr\_constrain | ( | size\_t | *K* | ) |  |

inline

Return the next Cholesky factor for a correlation matrix with the specified dimensionality, reading from an unconstrained vector of the appropriate size.

Parameters
:   |  |  |
    | --- | --- |
    | K | Rows and columns of Cholesky factor. |

Returns
:   Next Cholesky factor for a correlation matrix.

Exceptions
:   |  |  |
    | --- | --- |
    | std::domain\_error | if the matrix is not a valid Cholesky factor for a correlation matrix. |

Definition at line 1026 of file reader.hpp.

template<typename T >

|  |  |  |  |
| --- | --- | --- | --- |
| matrix\_t stan::io::reader< T >::cholesky\_corr\_constrain | ( | size\_t | *K*, |
|  |  | T & | *lp* |
|  | ) |  |  |

inline

Return the next Cholesky factor for a correlation matrix with the specified dimensionality, reading from an unconstrained vector of the appropriate size, and increment the log probability reference with the log Jacobian adjustment for the transform.

Parameters
:   |  |  |
    | --- | --- |
    | K | Rows and columns of Cholesky factor |

Returns
:   Next Cholesky factor for a correlation matrix.

Exceptions
:   |  |  |
    | --- | --- |
    | std::domain\_error | if the matrix is not a valid Cholesky factor for a correlation matrix. |

Definition at line 1043 of file reader.hpp.

template<typename T >

|  |  |  |  |
| --- | --- | --- | --- |
| matrix\_t stan::io::reader< T >::cholesky\_factor | ( | size\_t | *M*, |
|  |  | size\_t | *N* |
|  | ) |  |  |

inline

Return the next Cholesky factor with the specified dimensionality, reading it directly without transforms.

Parameters
:   |  |  |
    | --- | --- |
    | M | Rows of Cholesky factor |
    | N | Columns of Cholesky factor |

Returns
:   Next Cholesky factor.

Exceptions
:   |  |  |
    | --- | --- |
    | std::domain\_error | if the matrix is not a valid Cholesky factor. |

Definition at line 956 of file reader.hpp.

template<typename T >

|  |  |  |  |
| --- | --- | --- | --- |
| matrix\_t stan::io::reader< T >::cholesky\_factor\_constrain | ( | size\_t | *M*, |
|  |  | size\_t | *N* |
|  | ) |  |  |

inline

Return the next Cholesky factor with the specified dimensionality, reading from an unconstrained vector of the appropriate size.

Parameters
:   |  |  |
    | --- | --- |
    | M | Rows of Cholesky factor |
    | N | Columns of Cholesky factor |

Returns
:   Next Cholesky factor.

Exceptions
:   |  |  |
    | --- | --- |
    | std::domain\_error | if the matrix is not a valid Cholesky factor. |

Definition at line 975 of file reader.hpp.

template<typename T >

|  |  |  |  |
| --- | --- | --- | --- |
| matrix\_t stan::io::reader< T >::cholesky\_factor\_constrain | ( | size\_t | *M*, |
|  |  | size\_t | *N*, |
|  |  | T & | *lp* |
|  | ) |  |  |

inline

Return the next Cholesky factor with the specified dimensionality, reading from an unconstrained vector of the appropriate size, and increment the log probability reference with the log Jacobian adjustment for the transform.

Parameters
:   |  |  |  |
    | --- | --- | --- |
    |  | M | Rows of Cholesky factor |
    |  | N | Columns of Cholesky factor |
    | [in,out] | lp | log probability |

Returns
:   Next Cholesky factor.

Exceptions
:   |  |  |
    | --- | --- |
    | std::domain\_error | if the matrix is not a valid Cholesky factor. |

Definition at line 993 of file reader.hpp.

template<typename T >

|  |  |  |  |  |
| --- | --- | --- | --- | --- |
| T stan::io::reader< T >::corr | ( |  | ) |  |

inline

Return the next scalar, checking that it is a valid value for a correlation, between -1 (inclusive) and 1 (inclusive).

See `stan::math::check_bounded(T)`.

Returns
:   Next correlation value.

Exceptions
:   |  |  |
    | --- | --- |
    | std::runtime\_error | if the value is not valid for a correlation |

Definition at line 724 of file reader.hpp.

template<typename T >

|  |  |  |  |  |
| --- | --- | --- | --- | --- |
| T stan::io::reader< T >::corr\_constrain | ( |  | ) |  |

inline

Return the next scalar transformed to be a correlation between -1 and 1.

See `stan::prob::corr_constrain(T)`.

Returns
:   The next scalar transformed to a correlation.

Definition at line 739 of file reader.hpp.

template<typename T >

|  |  |  |  |  |  |
| --- | --- | --- | --- | --- | --- |
| T stan::io::reader< T >::corr\_constrain | ( | T & | *lp* | ) |  |

inline

Return the next scalar transformed to be a (partial) correlation between -1 and 1, incrementing the specified reference with the log of the absolute Jacobian determinant.

See `stan::prob::corr_constrain(T,T&)`.

Parameters
:   |  |  |
    | --- | --- |
    | lp | The reference to the variable holding the log probability to increment. |

Returns
:   The next scalar transformed to a correlation.

Definition at line 754 of file reader.hpp.

template<typename T >

|  |  |  |  |  |  |
| --- | --- | --- | --- | --- | --- |
| matrix\_t stan::io::reader< T >::corr\_matrix | ( | size\_t | *k* | ) |  |

inline

Returns the next correlation matrix of the specified dimensionality.

See `stan::math::check_corr_matrix(Matrix)`.

Parameters
:   |  |  |
    | --- | --- |
    | k | Dimensionality of correlation matrix. |

Returns
:   Next correlation matrix of the specified dimensionality.

Exceptions
:   |  |  |
    | --- | --- |
    | std::runtime\_error | if the matrix is not a correlation matrix |

Definition at line 1107 of file reader.hpp.

template<typename T >

|  |  |  |  |  |  |
| --- | --- | --- | --- | --- | --- |
| matrix\_t stan::io::reader< T >::corr\_matrix\_constrain | ( | size\_t | *k* | ) |  |

inline

Return the next correlation matrix of the specified dimensionality.

See `stan::prob::corr_matrix_constrain(Matrix)`.

Parameters
:   |  |  |
    | --- | --- |
    | k | Dimensionality of correlation matrix. |

Returns
:   Next correlation matrix of the specified dimensionality.

Definition at line 1123 of file reader.hpp.

template<typename T >

|  |  |  |  |
| --- | --- | --- | --- |
| matrix\_t stan::io::reader< T >::corr\_matrix\_constrain | ( | size\_t | *k*, |
|  |  | T & | *lp* |
|  | ) |  |  |

inline

Return the next correlation matrix of the specified dimensionality, incrementing the specified reference with the log absolute Jacobian determinant.

See `stan::prob::corr_matrix_constrain(Matrix,T&)`.

Parameters
:   |  |  |
    | --- | --- |
    | k | Dimensionality of the (square) correlation matrix. |
    | lp | Log probability reference to increment. |

Returns
:   The next correlation matrix of the specified dimensionality.

Definition at line 1138 of file reader.hpp.

template<typename T >

|  |  |  |  |  |  |
| --- | --- | --- | --- | --- | --- |
| matrix\_t stan::io::reader< T >::cov\_matrix | ( | size\_t | *k* | ) |  |

inline

Return the next covariance matrix with the specified dimensionality.

See `stan::math::check_cov_matrix(Matrix)`.

Parameters
:   |  |  |
    | --- | --- |
    | k | Dimensionality of covariance matrix. |

Returns
:   Next covariance matrix of the specified dimensionality.

Exceptions
:   |  |  |
    | --- | --- |
    | std::runtime\_error | if the matrix is not a valid covariance matrix |

Definition at line 1061 of file reader.hpp.

template<typename T >

|  |  |  |  |  |  |
| --- | --- | --- | --- | --- | --- |
| matrix\_t stan::io::reader< T >::cov\_matrix\_constrain | ( | size\_t | *k* | ) |  |

inline

Return the next covariance matrix of the specified dimensionality.

See `stan::prob::cov_matrix_constrain(Matrix)`.

Parameters
:   |  |  |
    | --- | --- |
    | k | Dimensionality of covariance matrix. |

Returns
:   Next covariance matrix of the specified dimensionality.

Definition at line 1076 of file reader.hpp.

template<typename T >

|  |  |  |  |
| --- | --- | --- | --- |
| matrix\_t stan::io::reader< T >::cov\_matrix\_constrain | ( | size\_t | *k*, |
|  |  | T & | *lp* |
|  | ) |  |  |

inline

Return the next covariance matrix of the specified dimensionality, incrementing the specified reference with the log absolute Jacobian determinant.

See `stan::prob::cov_matrix_constrain(Matrix,T&)`.

Parameters
:   |  |  |
    | --- | --- |
    | k | Dimensionality of the (square) covariance matrix. |
    | lp | Log probability reference to increment. |

Returns
:   The next covariance matrix of the specified dimensionality.

Definition at line 1092 of file reader.hpp.

template<typename T >

|  |  |  |  |  |
| --- | --- | --- | --- | --- |
| int stan::io::reader< T >::integer | ( |  | ) |  |

inline

Return the next integer in the integer sequence.

Returns
:   Next integer value.

Definition at line 119 of file reader.hpp.

template<typename T >

|  |  |  |  |  |
| --- | --- | --- | --- | --- |
| int stan::io::reader< T >::integer\_constrain | ( |  | ) |  |

inline

Return the next integer in the integer sequence.

This form is a convenience method to make compiling easier; its behavior is the same as `int()`

Returns
:   Next integer value.

Definition at line 133 of file reader.hpp.

template<typename T >

|  |  |  |  |  |  |
| --- | --- | --- | --- | --- | --- |
| int stan::io::reader< T >::integer\_constrain | ( | T & |  | ) |  |

inline

Return the next integer in the integer sequence.

This form is a convenience method to make compiling easier; its behavior is the same as `integer()`

Returns
:   Next integer value.

Definition at line 144 of file reader.hpp.

template<typename T >

|  |  |  |  |  |  |
| --- | --- | --- | --- | --- | --- |
| int stan::io::reader< T >::integer\_lb | ( | int | *lb* | ) |  |

inline

Return the next integer, checking that it is greater than or equal to the specified lower bound.

Parameters
:   |  |  |
    | --- | --- |
    | lb | Lower bound. |

Returns
:   Next integer read.

Exceptions
:   |  |  |
    | --- | --- |
    | std::runtime\_error | If the next integer read is not greater than or equal to the lower bound. |

Definition at line 331 of file reader.hpp.

template<typename T >

|  |  |  |  |  |  |
| --- | --- | --- | --- | --- | --- |
| int stan::io::reader< T >::integer\_lb\_constrain | ( | int | *lb* | ) |  |

inline

Return the next integer, checking that it is greater than or equal to the specified lower bound.

Parameters
:   |  |  |
    | --- | --- |
    | lb | Lower bound. |

Returns
:   Next integer read.

Exceptions
:   |  |  |
    | --- | --- |
    | std::runtime\_error | If the next integer read is not greater than or equal to the lower bound. |

Definition at line 347 of file reader.hpp.

template<typename T >

|  |  |  |  |
| --- | --- | --- | --- |
| int stan::io::reader< T >::integer\_lb\_constrain | ( | int | *lb*, |
|  |  | T & |  |
|  | ) |  |  |

inline

Return the next integer, checking that it is greater than or equal to the specified lower bound.

Parameters
:   |  |  |
    | --- | --- |
    | lb | Lower bound. lp Log probability (ignored because no Jacobian) |

Returns
:   Next integer read.

Exceptions
:   |  |  |
    | --- | --- |
    | std::runtime\_error | If the next integer read is not greater than or equal to the lower bound. |

Definition at line 360 of file reader.hpp.

template<typename T >

|  |  |  |  |
| --- | --- | --- | --- |
| int stan::io::reader< T >::integer\_lub | ( | int | *lb*, |
|  |  | int | *ub* |
|  | ) |  |  |

inline

Return the next integer, checking that it is less than or equal to the specified upper bound.

Even if the upper bounds and lower bounds are not consistent, the next integer value will be consumed.

Parameters
:   |  |  |
    | --- | --- |
    | lb | Lower bound. |
    | ub | Upper bound. |

Returns
:   Next integer read.

Exceptions
:   |  |  |
    | --- | --- |
    | std::runtime\_error | If the next integer read is not less than or equal to the upper bound. |

Definition at line 419 of file reader.hpp.

template<typename T >

|  |  |  |  |
| --- | --- | --- | --- |
| int stan::io::reader< T >::integer\_lub\_constrain | ( | int | *lb*, |
|  |  | int | *ub* |
|  | ) |  |  |

inline

Return the next integer, checking that it is less than or equal to the specified upper bound.

Parameters
:   |  |  |
    | --- | --- |
    | lb | Lower bound. |
    | ub | Upper bound. |

Returns
:   Next integer read.

Exceptions
:   |  |  |
    | --- | --- |
    | std::runtime\_error | If the next integer read is not less than or equal to the upper bound. |

Definition at line 443 of file reader.hpp.

template<typename T >

|  |  |  |  |
| --- | --- | --- | --- |
| int stan::io::reader< T >::integer\_lub\_constrain | ( | int | *lb*, |
|  |  | int | *ub*, |
|  |  | T & |  |
|  | ) |  |  |

inline

Return the next integer, checking that it is less than or equal to the specified upper bound.

Parameters
:   |  |  |
    | --- | --- |
    | lb | Lower bound. |
    | ub | Upper bound. lp Log probability (ignored because no Jacobian) |

Returns
:   Next integer read.

Exceptions
:   |  |  |
    | --- | --- |
    | std::runtime\_error | If the next integer read is not less than or equal to the upper bound. |

Definition at line 457 of file reader.hpp.

template<typename T >

|  |  |  |  |  |  |
| --- | --- | --- | --- | --- | --- |
| int stan::io::reader< T >::integer\_ub | ( | int | *ub* | ) |  |

inline

Return the next integer, checking that it is less than or equal to the specified upper bound.

Parameters
:   |  |  |
    | --- | --- |
    | ub | Upper bound. |

Returns
:   Next integer read.

Exceptions
:   |  |  |
    | --- | --- |
    | std::runtime\_error | If the next integer read is not less than or equal to the upper bound. |

Definition at line 374 of file reader.hpp.

template<typename T >

|  |  |  |  |  |  |
| --- | --- | --- | --- | --- | --- |
| int stan::io::reader< T >::integer\_ub\_constrain | ( | int | *ub* | ) |  |

inline

Return the next integer, checking that it is less than or equal to the specified upper bound.

Parameters
:   |  |  |
    | --- | --- |
    | ub | Upper bound. |

Returns
:   Next integer read.

Exceptions
:   |  |  |
    | --- | --- |
    | std::runtime\_error | If the next integer read is not less than or equal to the upper bound. |

Definition at line 390 of file reader.hpp.

template<typename T >

|  |  |  |  |
| --- | --- | --- | --- |
| int stan::io::reader< T >::integer\_ub\_constrain | ( | int | *ub*, |
|  |  | T & |  |
|  | ) |  |  |

inline

Return the next integer, checking that it is less than or equal to the specified upper bound.

Parameters
:   |  |  |
    | --- | --- |
    | ub | Upper bound. lp Log probability (ignored because no Jacobian) |

Returns
:   Next integer read.

Exceptions
:   |  |  |
    | --- | --- |
    | std::runtime\_error | If the next integer read is not less than or equal to the upper bound. |

Definition at line 403 of file reader.hpp.

template<typename T >

|  |  |  |  |
| --- | --- | --- | --- |
| matrix\_t stan::io::reader< T >::matrix | ( | size\_t | *m*, |
|  |  | size\_t | *n* |
|  | ) |  |  |

inline

Return a matrix of the specified dimensionality made up of the next scalars arranged in column-major order.

Row-major reading means that if a matrix of `m=2` rows and `n=3` columns is reada and the next scalar values are `1,2,3,4,5,6`, the result is

```
a = 1 4
    2 5
    3 6
```

Parameters
:   |  |  |
    | --- | --- |
    | m | Number of rows. |
    | n | Number of columns. |

Returns
:   Eigen::Matrix made up of the next scalars.

Definition at line 287 of file reader.hpp.

template<typename T >

|  |  |  |  |
| --- | --- | --- | --- |
| matrix\_t stan::io::reader< T >::matrix\_constrain | ( | size\_t | *m*, |
|  |  | size\_t | *n* |
|  | ) |  |  |

inline

Return a matrix of the specified dimensionality made up of the next scalars arranged in column-major order.

The constraint is a no-op. See `matrix(size_t, size_t)` for more information.

Parameters
:   |  |  |
    | --- | --- |
    | m | Number of rows. |
    | n | Number of columns. |

Returns
:   Matrix made up of the next scalars.

Definition at line 301 of file reader.hpp.

template<typename T >

|  |  |  |  |
| --- | --- | --- | --- |
| matrix\_t stan::io::reader< T >::matrix\_constrain | ( | size\_t | *m*, |
|  |  | size\_t | *n*, |
|  |  | T & |  |
|  | ) |  |  |

inline

Return a matrix of the specified dimensionality made up of the next scalars arranged in column-major order.

The constraint is a no-op, hence the log probability is not incremented. See `matrix(size_t, size_t)` for more information.

Parameters
:   |  |  |
    | --- | --- |
    | m | Number of rows. |
    | n | Number of columns. lp Log probability to increment. |

Returns
:   Matrix made up of the next scalars.

Definition at line 317 of file reader.hpp.

template<typename T >

template<typename TL >

|  |  |  |  |
| --- | --- | --- | --- |
| matrix\_t stan::io::reader< T >::matrix\_lb | ( | const TL | *lb*, |
|  |  | size\_t | *m*, |
|  |  | size\_t | *n* |
|  | ) |  |  |

inline

Definition at line 1188 of file reader.hpp.

template<typename T >

template<typename TL >

|  |  |  |  |
| --- | --- | --- | --- |
| matrix\_t stan::io::reader< T >::matrix\_lb\_constrain | ( | const TL | *lb*, |
|  |  | size\_t | *m*, |
|  |  | size\_t | *n* |
|  | ) |  |  |

inline

Definition at line 1196 of file reader.hpp.

template<typename T >

template<typename TL >

|  |  |  |  |
| --- | --- | --- | --- |
| matrix\_t stan::io::reader< T >::matrix\_lb\_constrain | ( | const TL | *lb*, |
|  |  | size\_t | *m*, |
|  |  | size\_t | *n*, |
|  |  | T & | *lp* |
|  | ) |  |  |

inline

Definition at line 1204 of file reader.hpp.

template<typename T >

template<typename TL , typename TU >

|  |  |  |  |
| --- | --- | --- | --- |
| matrix\_t stan::io::reader< T >::matrix\_lub | ( | const TL | *lb*, |
|  |  | const TU | *ub*, |
|  |  | size\_t | *m*, |
|  |  | size\_t | *n* |
|  | ) |  |  |

inline

Definition at line 1327 of file reader.hpp.

template<typename T >

template<typename TL , typename TU >

|  |  |  |  |
| --- | --- | --- | --- |
| matrix\_t stan::io::reader< T >::matrix\_lub\_constrain | ( | const TL | *lb*, |
|  |  | const TU | *ub*, |
|  |  | size\_t | *m*, |
|  |  | size\_t | *n* |
|  | ) |  |  |

inline

Definition at line 1335 of file reader.hpp.

template<typename T >

template<typename TL , typename TU >

|  |  |  |  |
| --- | --- | --- | --- |
| matrix\_t stan::io::reader< T >::matrix\_lub\_constrain | ( | const TL | *lb*, |
|  |  | const TU | *ub*, |
|  |  | size\_t | *m*, |
|  |  | size\_t | *n*, |
|  |  | T & | *lp* |
|  | ) |  |  |

inline

Definition at line 1343 of file reader.hpp.

template<typename T >

template<typename TU >

|  |  |  |  |
| --- | --- | --- | --- |
| matrix\_t stan::io::reader< T >::matrix\_ub | ( | const TU | *ub*, |
|  |  | size\_t | *m*, |
|  |  | size\_t | *n* |
|  | ) |  |  |

inline

Definition at line 1257 of file reader.hpp.

template<typename T >

template<typename TU >

|  |  |  |  |
| --- | --- | --- | --- |
| matrix\_t stan::io::reader< T >::matrix\_ub\_constrain | ( | const TU | *ub*, |
|  |  | size\_t | *m*, |
|  |  | size\_t | *n* |
|  | ) |  |  |

inline

Definition at line 1265 of file reader.hpp.

template<typename T >

template<typename TU >

|  |  |  |  |
| --- | --- | --- | --- |
| matrix\_t stan::io::reader< T >::matrix\_ub\_constrain | ( | const TU | *ub*, |
|  |  | size\_t | *m*, |
|  |  | size\_t | *n*, |
|  |  | T & | *lp* |
|  | ) |  |  |

inline

Definition at line 1273 of file reader.hpp.

template<typename T >

|  |  |  |  |  |  |
| --- | --- | --- | --- | --- | --- |
| vector\_t stan::io::reader< T >::ordered | ( | size\_t | *k* | ) |  |

inline

Return the next vector of specified size containing values in ascending order.

See `stan::math::check_ordered(T)` for behavior on failure.

Parameters
:   |  |  |
    | --- | --- |
    | k | Size of returned vector. |

Returns
:   Vector of positive values in ascending order.

Definition at line 864 of file reader.hpp.

template<typename T >

|  |  |  |  |  |  |
| --- | --- | --- | --- | --- | --- |
| vector\_t stan::io::reader< T >::ordered\_constrain | ( | size\_t | *k* | ) |  |

inline

Return the next ordered vector of the specified length.

See `stan::prob::ordered_constrain(Matrix)`.

Parameters
:   |  |  |
    | --- | --- |
    | k | Length of returned vector. |

Returns
:   Next ordered vector of the specified length.

Definition at line 879 of file reader.hpp.

template<typename T >

|  |  |  |  |
| --- | --- | --- | --- |
| vector\_t stan::io::reader< T >::ordered\_constrain | ( | size\_t | *k*, |
|  |  | T & | *lp* |
|  | ) |  |  |

inline

Return the next ordered vector of the specified size, incrementing the specified reference with the log absolute Jacobian of the determinant.

See `stan::prob::ordered_constrain(Matrix,T&)`.

Parameters
:   |  |  |
    | --- | --- |
    | k | Size of vector. |
    | lp | Log probability reference to increment. |

Returns
:   Next ordered vector of the specified size.

Definition at line 894 of file reader.hpp.

template<typename T >

|  |  |  |  |  |  |
| --- | --- | --- | --- | --- | --- |
| vector\_t stan::io::reader< T >::positive\_ordered | ( | size\_t | *k* | ) |  |

inline

Return the next vector of specified size containing positive values in ascending order.

See `stan::math::check_positive_ordered(T)` for behavior on failure.

Parameters
:   |  |  |
    | --- | --- |
    | k | Size of returned vector. |

Returns
:   Vector of positive values in ascending order.

Definition at line 908 of file reader.hpp.

template<typename T >

|  |  |  |  |  |  |
| --- | --- | --- | --- | --- | --- |
| vector\_t stan::io::reader< T >::positive\_ordered\_constrain | ( | size\_t | *k* | ) |  |

inline

Return the next positive ordered vector of the specified length.

See `stan::prob::positive_ordered_constrain(Matrix)`.

Parameters
:   |  |  |
    | --- | --- |
    | k | Length of returned vector. |

Returns
:   Next positive\_ordered vector of the specified length.

Definition at line 925 of file reader.hpp.

template<typename T >

|  |  |  |  |
| --- | --- | --- | --- |
| vector\_t stan::io::reader< T >::positive\_ordered\_constrain | ( | size\_t | *k*, |
|  |  | T & | *lp* |
|  | ) |  |  |

inline

Return the next positive\_ordered vector of the specified size, incrementing the specified reference with the log absolute Jacobian of the determinant.

See `stan::prob::positive_ordered_constrain(Matrix,T&)`.

Parameters
:   |  |  |
    | --- | --- |
    | k | Size of vector. |
    | lp | Log probability reference to increment. |

Returns
:   Next positive\_ordered vector of the specified size.

Definition at line 940 of file reader.hpp.

template<typename T >

|  |  |  |  |  |
| --- | --- | --- | --- | --- |
| T stan::io::reader< T >::prob | ( |  | ) |  |

inline

Return the next scalar, checking that it is a valid value for a probability, between 0 (inclusive) and 1 (inclusive).

See `stan::math::check_bounded(T)`.

Returns
:   Next probability value.

Definition at line 677 of file reader.hpp.

template<typename T >

|  |  |  |  |  |
| --- | --- | --- | --- | --- |
| T stan::io::reader< T >::prob\_constrain | ( |  | ) |  |

inline

Return the next scalar transformed to be a probability between 0 and 1.

See `stan::prob::prob_constrain(T)`.

Returns
:   The next scalar transformed to a probability.

Definition at line 692 of file reader.hpp.

template<typename T >

|  |  |  |  |  |  |
| --- | --- | --- | --- | --- | --- |
| T stan::io::reader< T >::prob\_constrain | ( | T & | *lp* | ) |  |

inline

Return the next scalar transformed to be a probability between 0 and 1, incrementing the specified reference with the log of the absolute Jacobian determinant.

See `stan::prob::prob_constrain(T)`.

Parameters
:   |  |  |
    | --- | --- |
    | lp | Reference to log probability variable to increment. |

Returns
:   The next scalar transformed to a probability.

Definition at line 706 of file reader.hpp.

template<typename T >

|  |  |  |  |  |  |
| --- | --- | --- | --- | --- | --- |
| row\_vector\_t stan::io::reader< T >::row\_vector | ( | size\_t | *m* | ) |  |

inline

Return a row vector of specified dimensionality made up of the next scalars.

Parameters
:   |  |  |
    | --- | --- |
    | m | Number of rows in the vector to read. |

Returns
:   Column vector made up of the next scalars.

Definition at line 242 of file reader.hpp.

template<typename T >

|  |  |  |  |  |  |
| --- | --- | --- | --- | --- | --- |
| row\_vector\_t stan::io::reader< T >::row\_vector\_constrain | ( | size\_t | *m* | ) |  |

inline

Return a row vector of specified dimensionality made up of the next scalars.

The constraint is a no-op.

Parameters
:   |  |  |
    | --- | --- |
    | m | Number of rows in the vector to read. |

Returns
:   Column vector made up of the next scalars.

Definition at line 253 of file reader.hpp.

template<typename T >

|  |  |  |  |
| --- | --- | --- | --- |
| row\_vector\_t stan::io::reader< T >::row\_vector\_constrain | ( | size\_t | *m*, |
|  |  | T & |  |
|  | ) |  |  |

inline

Return a row vector of specified dimensionality made up of the next scalars.

The constraint is a no-op, so the log probability is not incremented.

Parameters
:   |  |  |
    | --- | --- |
    | m | Number of rows in the vector to read. lp Log probability to increment. |

Returns
:   Column vector made up of the next scalars.

Definition at line 266 of file reader.hpp.

template<typename T >

template<typename TL >

|  |  |  |  |
| --- | --- | --- | --- |
| row\_vector\_t stan::io::reader< T >::row\_vector\_lb | ( | const TL | *lb*, |
|  |  | size\_t | *m* |
|  | ) |  |  |

inline

Definition at line 1166 of file reader.hpp.

template<typename T >

template<typename TL >

|  |  |  |  |
| --- | --- | --- | --- |
| row\_vector\_t stan::io::reader< T >::row\_vector\_lb\_constrain | ( | const TL | *lb*, |
|  |  | size\_t | *m* |
|  | ) |  |  |

inline

Definition at line 1173 of file reader.hpp.

template<typename T >

template<typename TL >

|  |  |  |  |
| --- | --- | --- | --- |
| row\_vector\_t stan::io::reader< T >::row\_vector\_lb\_constrain | ( | const TL | *lb*, |
|  |  | size\_t | *m*, |
|  |  | T & | *lp* |
|  | ) |  |  |

inline

Definition at line 1180 of file reader.hpp.

template<typename T >

template<typename TL , typename TU >

|  |  |  |  |
| --- | --- | --- | --- |
| row\_vector\_t stan::io::reader< T >::row\_vector\_lub | ( | const TL | *lb*, |
|  |  | const TU | *ub*, |
|  |  | size\_t | *m* |
|  | ) |  |  |

inline

Definition at line 1305 of file reader.hpp.

template<typename T >

template<typename TL , typename TU >

|  |  |  |  |
| --- | --- | --- | --- |
| row\_vector\_t stan::io::reader< T >::row\_vector\_lub\_constrain | ( | const TL | *lb*, |
|  |  | const TU | *ub*, |
|  |  | size\_t | *m* |
|  | ) |  |  |

inline

Definition at line 1312 of file reader.hpp.

template<typename T >

template<typename TL , typename TU >

|  |  |  |  |
| --- | --- | --- | --- |
| row\_vector\_t stan::io::reader< T >::row\_vector\_lub\_constrain | ( | const TL | *lb*, |
|  |  | const TU | *ub*, |
|  |  | size\_t | *m*, |
|  |  | T & | *lp* |
|  | ) |  |  |

inline

Definition at line 1319 of file reader.hpp.

template<typename T >

template<typename TU >

|  |  |  |  |
| --- | --- | --- | --- |
| row\_vector\_t stan::io::reader< T >::row\_vector\_ub | ( | const TU | *ub*, |
|  |  | size\_t | *m* |
|  | ) |  |  |

inline

Definition at line 1235 of file reader.hpp.

template<typename T >

template<typename TU >

|  |  |  |  |
| --- | --- | --- | --- |
| row\_vector\_t stan::io::reader< T >::row\_vector\_ub\_constrain | ( | const TU | *ub*, |
|  |  | size\_t | *m* |
|  | ) |  |  |

inline

Definition at line 1242 of file reader.hpp.

template<typename T >

template<typename TU >

|  |  |  |  |
| --- | --- | --- | --- |
| row\_vector\_t stan::io::reader< T >::row\_vector\_ub\_constrain | ( | const TU | *ub*, |
|  |  | size\_t | *m*, |
|  |  | T & | *lp* |
|  | ) |  |  |

inline

Definition at line 1249 of file reader.hpp.

template<typename T >

|  |  |  |  |  |
| --- | --- | --- | --- | --- |
| T stan::io::reader< T >::scalar | ( |  | ) |  |

inline

Return the next scalar in the sequence.

Returns
:   Next scalar value.

Definition at line 155 of file reader.hpp.

template<typename T >

|  |  |  |  |  |
| --- | --- | --- | --- | --- |
| T stan::io::reader< T >::scalar\_constrain | ( |  | ) |  |

inline

Return the next scalar.

For arbitrary scalars, constraint is a no-op.

Returns
:   Next scalar.

Definition at line 167 of file reader.hpp.

template<typename T >

|  |  |  |  |  |  |
| --- | --- | --- | --- | --- | --- |
| T stan::io::reader< T >::scalar\_constrain | ( | T & |  | ) |  |

inline

Return the next scalar in the sequence, incrementing the specified reference with the log absolute Jacobian determinant.

With no transformation, the Jacobian increment is a no-op.

See `scalar_constrain()`.

log\_prob Reference to log probability variable to increment.

Returns
:   Next scalar.

Definition at line 182 of file reader.hpp.

template<typename T >

template<typename TL >

|  |  |  |  |  |  |
| --- | --- | --- | --- | --- | --- |
| T stan::io::reader< T >::scalar\_lb | ( | const TL | *lb* | ) |  |

inline

Return the next scalar, checking that it is greater than or equal to the specified lower bound.

See `stan::math::check_greater_or_equal(T,double)`.

Parameters
:   |  |  |
    | --- | --- |
    | lb | Lower bound. |

Returns
:   Next scalar value.

Template Parameters
:   |  |  |
    | --- | --- |
    | TL | Type of lower bound. |

Exceptions
:   |  |  |
    | --- | --- |
    | std::runtime\_error | if the scalar is less than the specified lower bound |

Definition at line 517 of file reader.hpp.

template<typename T >

template<typename TL >

|  |  |  |  |  |  |
| --- | --- | --- | --- | --- | --- |
| T stan::io::reader< T >::scalar\_lb\_constrain | ( | const TL | *lb* | ) |  |

inline

Return the next scalar transformed to have the specified lower bound.

See `stan::prob::lb_constrain(T,double)`.

Template Parameters
:   |  |  |
    | --- | --- |
    | TL | Type of lower bound. |

Parameters
:   |  |  |
    | --- | --- |
    | lb | Lower bound on values. |

Returns
:   Next scalar transformed to have the specified lower bound.

Definition at line 537 of file reader.hpp.

template<typename T >

template<typename TL >

|  |  |  |  |
| --- | --- | --- | --- |
| T stan::io::reader< T >::scalar\_lb\_constrain | ( | const TL | *lb*, |
|  |  | T & | *lp* |
|  | ) |  |  |

inline

Return the next scalar transformed to have the specified lower bound, incrementing the specified reference with the log of the absolute Jacobian determinant of the transform.

See `stan::prob::lb_constrain(T,double,T&)`.

Template Parameters
:   |  |  |
    | --- | --- |
    | TL | Type of lower bound. |

Parameters
:   |  |  |
    | --- | --- |
    | lb | Lower bound on result. |
    | lp | Reference to log probability variable to increment. |

Definition at line 553 of file reader.hpp.

template<typename T >

template<typename TL , typename TU >

|  |  |  |  |
| --- | --- | --- | --- |
| T stan::io::reader< T >::scalar\_lub | ( | const TL | *lb*, |
|  |  | const TU | *ub* |
|  | ) |  |  |

inline

Return the next scalar, checking that it is between the specified lower and upper bound.

See `stan::math::check_bounded(T,double,double)`.

Template Parameters
:   |  |  |
    | --- | --- |
    | TL | Type of lower bound. |
    | TU | Type of upper bound. |

Parameters
:   |  |  |
    | --- | --- |
    | lb | Lower bound. |
    | ub | Upper bound. |

Returns
:   Next scalar value.

Exceptions
:   |  |  |
    | --- | --- |
    | std::runtime\_error | if the scalar is not between the specified lower and upper bounds. |

Definition at line 626 of file reader.hpp.

template<typename T >

template<typename TL , typename TU >

|  |  |  |  |
| --- | --- | --- | --- |
| T stan::io::reader< T >::scalar\_lub\_constrain | ( | const TL | *lb*, |
|  |  | const TU | *ub* |
|  | ) |  |  |

inline

Return the next scalar transformed to be between the specified lower and upper bounds.

See `stan::prob::lub_constrain(T,double,double)`.

Template Parameters
:   |  |  |
    | --- | --- |
    | TL | Type of lower bound. |
    | TU | Type of upper bound. |

Parameters
:   |  |  |
    | --- | --- |
    | lb | Lower bound. |
    | ub | Upper bound. |

Returns
:   Next scalar transformed to fall between the specified bounds.

Definition at line 647 of file reader.hpp.

template<typename T >

template<typename TL , typename TU >

|  |  |  |  |
| --- | --- | --- | --- |
| T stan::io::reader< T >::scalar\_lub\_constrain | ( | TL | *lb*, |
|  |  | TU | *ub*, |
|  |  | T & | *lp* |
|  | ) |  |  |

inline

Return the next scalar transformed to be between the the specified lower and upper bounds.

See `stan::prob::lub_constrain(T,double,double,T&)`.

Parameters
:   |  |  |
    | --- | --- |
    | lb | Lower bound. |
    | ub | Upper bound. |
    | lp | Reference to log probability variable to increment. |

Template Parameters
:   |  |  |
    | --- | --- |
    | T | Type of scalar. |
    | TL | Type of lower bound. |
    | TU | Type of upper bound. |

Definition at line 665 of file reader.hpp.

template<typename T >

|  |  |  |  |  |
| --- | --- | --- | --- | --- |
| T stan::io::reader< T >::scalar\_pos | ( |  | ) |  |

inline

Return the next scalar, checking that it is positive.

See `stan::math::check_positive(T)`.

Returns
:   Next positive scalar.

Exceptions
:   |  |  |
    | --- | --- |
    | std::runtime\_error | if x is not positive |

Definition at line 472 of file reader.hpp.

template<typename T >

|  |  |  |  |  |
| --- | --- | --- | --- | --- |
| T stan::io::reader< T >::scalar\_pos\_constrain | ( |  | ) |  |

inline

Return the next scalar, transformed to be positive.

See `stan::prob::positive_constrain(T)`.

Returns
:   The next scalar transformed to be positive.

Definition at line 486 of file reader.hpp.

template<typename T >

|  |  |  |  |  |  |
| --- | --- | --- | --- | --- | --- |
| T stan::io::reader< T >::scalar\_pos\_constrain | ( | T & | *lp* | ) |  |

inline

Return the next scalar transformed to be positive, incrementing the specified reference with the log absolute determinant of the Jacobian.

See `stan::prob::positive_constrain(T,T&)`.

Parameters
:   |  |  |
    | --- | --- |
    | lp | Reference to log probability variable to increment. |

Returns
:   The next scalar transformed to be positive.

Definition at line 500 of file reader.hpp.

template<typename T >

template<typename TU >

|  |  |  |  |  |  |
| --- | --- | --- | --- | --- | --- |
| T stan::io::reader< T >::scalar\_ub | ( | TU | *ub* | ) |  |

inline

Return the next scalar, checking that it is less than or equal to the specified upper bound.

See `stan::math::check_less_or_equal(T,double)`.

Template Parameters
:   |  |  |
    | --- | --- |
    | TU | Type of upper bound. |

Parameters
:   |  |  |
    | --- | --- |
    | ub | Upper bound. |

Returns
:   Next scalar value.

Exceptions
:   |  |  |
    | --- | --- |
    | std::runtime\_error | if the scalar is greater than the specified upper bound |

Definition at line 572 of file reader.hpp.

template<typename T >

template<typename TU >

|  |  |  |  |  |  |
| --- | --- | --- | --- | --- | --- |
| T stan::io::reader< T >::scalar\_ub\_constrain | ( | const TU | *ub* | ) |  |

inline

Return the next scalar transformed to have the specified upper bound.

See `stan::prob::ub_constrain(T,double)`.

Template Parameters
:   |  |  |
    | --- | --- |
    | TU | Type of upper bound. |

Parameters
:   |  |  |
    | --- | --- |
    | ub | Upper bound on values. |

Returns
:   Next scalar transformed to have the specified upper bound.

Definition at line 591 of file reader.hpp.

template<typename T >

template<typename TU >

|  |  |  |  |
| --- | --- | --- | --- |
| T stan::io::reader< T >::scalar\_ub\_constrain | ( | const TU | *ub*, |
|  |  | T & | *lp* |
|  | ) |  |  |

inline

Return the next scalar transformed to have the specified upper bound, incrementing the specified reference with the log of the absolute Jacobian determinant of the transform.

See `stan::prob::ub_constrain(T,double,T&)`.

Template Parameters
:   |  |  |
    | --- | --- |
    | TU | Type of upper bound. |

Parameters
:   |  |  |
    | --- | --- |
    | ub | Upper bound on result. |
    | lp | Reference to log probability variable to increment. |

Definition at line 607 of file reader.hpp.

template<typename T >

|  |  |  |  |  |  |
| --- | --- | --- | --- | --- | --- |
| vector\_t stan::io::reader< T >::simplex | ( | size\_t | *k* | ) |  |

inline

Return a simplex of the specified size made up of the next scalars.

See `stan::math::check_simplex`.

Parameters
:   |  |  |
    | --- | --- |
    | k | Size of returned simplex. |

Returns
:   Simplex read from the specified size number of scalars.

Exceptions
:   |  |  |
    | --- | --- |
    | std::runtime\_error | if the k values is not a simplex. |

Definition at line 816 of file reader.hpp.

template<typename T >

|  |  |  |  |  |  |
| --- | --- | --- | --- | --- | --- |
| Eigen::Matrix<T,Eigen::Dynamic,1> stan::io::reader< T >::simplex\_constrain | ( | size\_t | *k* | ) |  |

inline

Return the next simplex transformed vector of the specified length.

This operation consumes one less than the specified length number of scalars.

See `stan::prob::simplex_constrain(Eigen::Matrix)`.

Parameters
:   |  |  |
    | --- | --- |
    | k | Number of dimensions in resulting simplex. |

Returns
:   Simplex derived from next `k-1` scalars.

Definition at line 834 of file reader.hpp.

template<typename T >

|  |  |  |  |
| --- | --- | --- | --- |
| vector\_t stan::io::reader< T >::simplex\_constrain | ( | size\_t | *k*, |
|  |  | T & | *lp* |
|  | ) |  |  |

inline

Return the next simplex of the specified size (using one fewer unconstrained scalars), incrementing the specified reference with the log absolute Jacobian determinant.

See `stan::prob::simplex_constrain(Eigen::Matrix,T&)`.

Parameters
:   |  |  |
    | --- | --- |
    | k | Size of simplex. |
    | lp | Log probability to increment with log absolute Jacobian determinant. |

Returns
:   The next simplex of the specified size.

Definition at line 850 of file reader.hpp.

template<typename T >

|  |  |  |  |  |  |
| --- | --- | --- | --- | --- | --- |
| std::vector<T> stan::io::reader< T >::std\_vector | ( | size\_t | *m* | ) |  |

inline

Return a standard library vector of the specified dimensionality made up of the next scalars.

Parameters
:   |  |  |
    | --- | --- |
    | m | Size of vector. |

Returns
:   Vector made up of the next scalars.

Definition at line 194 of file reader.hpp.

template<typename T >

|  |  |  |  |  |  |
| --- | --- | --- | --- | --- | --- |
| vector\_t stan::io::reader< T >::unit\_vector | ( | size\_t | *k* | ) |  |

inline

Return a unit\_vector of the specified size made up of the next scalars.

See `stan::math::check_unit_vector`.

Parameters
:   |  |  |
    | --- | --- |
    | k | Size of returned unit\_vector. |

Returns
:   unit\_vector read from the specified size number of scalars.

Exceptions
:   |  |  |
    | --- | --- |
    | std::runtime\_error | if the k values is not a unit\_vector. |

Definition at line 768 of file reader.hpp.

template<typename T >

|  |  |  |  |  |  |
| --- | --- | --- | --- | --- | --- |
| Eigen::Matrix<T,Eigen::Dynamic,1> stan::io::reader< T >::unit\_vector\_constrain | ( | size\_t | *k* | ) |  |

inline

Return the next unit\_vector transformed vector of the specified length.

This operation consumes one less than the specified length number of scalars.

See `stan::prob::unit_vector_constrain(Eigen::Matrix)`.

Parameters
:   |  |  |
    | --- | --- |
    | k | Number of dimensions in resulting unit\_vector. |

Returns
:   unit\_vector derived from next `k-1` scalars.

Definition at line 786 of file reader.hpp.

template<typename T >

|  |  |  |  |
| --- | --- | --- | --- |
| vector\_t stan::io::reader< T >::unit\_vector\_constrain | ( | size\_t | *k*, |
|  |  | T & | *lp* |
|  | ) |  |  |

inline

Return the next unit\_vector of the specified size (using one fewer unconstrained scalars), incrementing the specified reference with the log absolute Jacobian determinant.

See `stan::prob::unit_vector_constrain(Eigen::Matrix,T&)`.

Parameters
:   |  |  |
    | --- | --- |
    | k | Size of unit\_vector. |
    | lp | Log probability to increment with log absolute Jacobian determinant. |

Returns
:   The next unit\_vector of the specified size.

Definition at line 802 of file reader.hpp.

template<typename T >

|  |  |  |  |  |  |
| --- | --- | --- | --- | --- | --- |
| vector\_t stan::io::reader< T >::vector | ( | size\_t | *m* | ) |  |

inline

Return a column vector of specified dimensionality made up of the next scalars.

Parameters
:   |  |  |
    | --- | --- |
    | m | Number of rows in the vector to read. |

Returns
:   Column vector made up of the next scalars.

Definition at line 208 of file reader.hpp.

template<typename T >

|  |  |  |  |  |  |
| --- | --- | --- | --- | --- | --- |
| vector\_t stan::io::reader< T >::vector\_constrain | ( | size\_t | *m* | ) |  |

inline

Return a column vector of specified dimensionality made up of the next scalars.

The constraint is a no-op.

Parameters
:   |  |  |
    | --- | --- |
    | m | Number of rows in the vector to read. |

Returns
:   Column vector made up of the next scalars.

Definition at line 218 of file reader.hpp.

template<typename T >

|  |  |  |  |
| --- | --- | --- | --- |
| vector\_t stan::io::reader< T >::vector\_constrain | ( | size\_t | *m*, |
|  |  | T & |  |
|  | ) |  |  |

inline

Return a column vector of specified dimensionality made up of the next scalars.

The constraint and hence Jacobian are no-ops.

Parameters
:   |  |  |
    | --- | --- |
    | m | Number of rows in the vector to read. lp Log probability to increment. |

Returns
:   Column vector made up of the next scalars.

Definition at line 229 of file reader.hpp.

template<typename T >

template<typename TL >

|  |  |  |  |
| --- | --- | --- | --- |
| vector\_t stan::io::reader< T >::vector\_lb | ( | const TL | *lb*, |
|  |  | size\_t | *m* |
|  | ) |  |  |

inline

Definition at line 1144 of file reader.hpp.

template<typename T >

template<typename TL >

|  |  |  |  |
| --- | --- | --- | --- |
| vector\_t stan::io::reader< T >::vector\_lb\_constrain | ( | const TL | *lb*, |
|  |  | size\_t | *m* |
|  | ) |  |  |

inline

Definition at line 1151 of file reader.hpp.

template<typename T >

template<typename TL >

|  |  |  |  |
| --- | --- | --- | --- |
| vector\_t stan::io::reader< T >::vector\_lb\_constrain | ( | const TL | *lb*, |
|  |  | size\_t | *m*, |
|  |  | T & | *lp* |
|  | ) |  |  |

inline

Definition at line 1158 of file reader.hpp.

template<typename T >

template<typename TL , typename TU >

|  |  |  |  |
| --- | --- | --- | --- |
| vector\_t stan::io::reader< T >::vector\_lub | ( | const TL | *lb*, |
|  |  | const TU | *ub*, |
|  |  | size\_t | *m* |
|  | ) |  |  |

inline

Definition at line 1283 of file reader.hpp.

template<typename T >

template<typename TL , typename TU >

|  |  |  |  |
| --- | --- | --- | --- |
| vector\_t stan::io::reader< T >::vector\_lub\_constrain | ( | const TL | *lb*, |
|  |  | const TU | *ub*, |
|  |  | size\_t | *m* |
|  | ) |  |  |

inline

Definition at line 1290 of file reader.hpp.

template<typename T >

template<typename TL , typename TU >

|  |  |  |  |
| --- | --- | --- | --- |
| vector\_t stan::io::reader< T >::vector\_lub\_constrain | ( | const TL | *lb*, |
|  |  | const TU | *ub*, |
|  |  | size\_t | *m*, |
|  |  | T & | *lp* |
|  | ) |  |  |

inline

Definition at line 1297 of file reader.hpp.

template<typename T >

template<typename TU >

|  |  |  |  |
| --- | --- | --- | --- |
| vector\_t stan::io::reader< T >::vector\_ub | ( | const TU | *ub*, |
|  |  | size\_t | *m* |
|  | ) |  |  |

inline

Definition at line 1213 of file reader.hpp.

template<typename T >

template<typename TU >

|  |  |  |  |
| --- | --- | --- | --- |
| vector\_t stan::io::reader< T >::vector\_ub\_constrain | ( | const TU | *ub*, |
|  |  | size\_t | *m* |
|  | ) |  |  |

inline

Definition at line 1220 of file reader.hpp.

template<typename T >

template<typename TU >

|  |  |  |  |
| --- | --- | --- | --- |
| vector\_t stan::io::reader< T >::vector\_ub\_constrain | ( | const TU | *ub*, |
|  |  | size\_t | *m*, |
|  |  | T & | *lp* |
|  | ) |  |  |

inline

Definition at line 1227 of file reader.hpp.

---

The documentation for this class was generated from the following file:

- src/stan/io/reader.hpp


---

[ Stan Home Page ]

*© 2011–2014,
Stan Development Team.*
